# Development of quality indicators for palliative care in intensive care units and pilot testing them via electronic medical record review

**DOI:** 10.1186/s40560-023-00713-z

**Published:** 2024-01-09

**Authors:** Yuta Tanaka, Kento Masukawa, Hideaki Sakuramoto, Akane Kato, Yuichiro Ishigami, Junko Tatsuno, Kaori Ito, Yoshiyuki Kizawa, Mitsunori Miyashita

**Affiliations:** 1 Department of Palliative Nursing, Health Sciences, Tohoku University Graduate School of Medicine, 2-1 Seiryo-Machi, Aoba-ku, Sendai, Miyagi 980-8575 Japan; 2 Department of Critical Care and Disaster Nursing, Japanese Red Cross Kyushu International College of Nursing, Munakata, Fukuoka Japan; 3 Department of Adult and Geriatric Nursing, School of Health Science, Shinshu University, Matsumoto, Nagano, Japan; 4 Department of Transitional and Palliative Care, Aso Iizuka Hospital, Fukuoka, Japan; 5 Nursing Department, Kokura Memorial Hospital, Fukuoka, Japan; 6 Department of Surgery, Division of Acute Care Surgery, Teikyo University School of Medicine, Tokyo, Japan; 7 Department of Palliative and Supportive Care, Institute of Medicine, University of Tsukuba, Tsukuba, Japan

**Keywords:** Quality indicator, Palliative care, Critical care, Intensive care, Intensive care units

## Abstract

**Background:**

Patients in intensive care units (ICUs) often require quality palliative care for relief from various types of suffering. To achieve quality palliative care, specific goals need to be identified, measured, and reported. The present study aimed to develop quality indicators (QIs) for palliative care in ICUs, based on a systematic review and modified Delphi method, and test their feasibility by reviewing electronic medical record (EMR) data.

**Methods:**

The current study was performed in two phases: the development of QIs using the modified Delphi method, and pilot-testing the quality of palliative care in ICUs based on EMR review. The pilot test included 262 patients admitted to the general or emergency ICU at a university hospital from January 1, 2019, to June 30, 2019.

**Results:**

A 28-item QI set for palliative care in ICUs was developed based on the consensus of 16 experts. The Delphi process resulted in low measurability ratings for two items: "Assessment of the patient's psychological distress" and "Assessment of the patient's spiritual and cultural practices." However, these items were determined to be important for quality care from the perspective of holistic assessment of distress and were adopted in the final version of the QI set. While the pilot test results indicated the feasibility of the developed QIs, they suggested that the frequency of care performance varied, and certain aspects of palliative care in ICUs needed to be improved, namely (1) regular pain assessment, (2) identification of the patient's advance directive and advance care planning for treatment, (3) conducting an interdisciplinary family conference on palliative care, and (4) assessment of psychological distress of family members.

**Conclusions:**

The QI set, developed using the modified Delphi method and tested using EMR data, provided a tool for assessing the quality of palliative care in ICUs. In the two ICUs considered in this study, aspects of the palliative care process with a low performance frequency were identified, and further national surveys were recommended. It is necessary to conduct ongoing surveys at more facilities to improve the quality of palliative care in ICUs.

**Supplementary Information:**

The online version contains supplementary material available at 10.1186/s40560-023-00713-z.

## Background

Many patients in intensive care units (ICUs) need palliative care [1–3] to improve their quality of life as well as that of their families as they all face physical, psychological, social, or spiritual challenges associated with life-threatening illnesses [4]. In ICUs, approximately 20–70% of patients experience physical distress, such as pain, dyspnea, thirst, and fatigue [5], whereas 30–60% experience psychological distress, such as anxiety, depression, and post-traumatic stress disorder [6, 7]. Additionally, patients experience social and spiritual distress, such as loss of social roles, fear of death, loneliness, and loss of self-control [8]. Besides the patients, their families also experience psychological distress, including anxiety and persistent grief disorder [9, 10]. Therefore, quality ICU care should provide palliative care. Basic palliative care for ICU patients can shorten ICU stays, reduce the use of non-beneficial life-sustaining therapies, and reduce psychological distress in patients' families, without shortening patients’ life expectancy [11, 12]. High-quality palliative care can improve the quality of life and symptoms of patients and their families, and may reduce healthcare costs by aligning care with end-of-life goals [11, 13–16].

Quality indicators (QIs) are statements that define the quality of a service explicitly and quantifiably [17]. Measuring and reporting quality of care using QIs can identify potential problems in care and serve as foundation for further improvement [18, 19]. Previous studies, mainly conducted in the United States, developed and evaluated QIs using electronic medical record (EMR) data [20–22]. EMR data have the advantage of reflecting the patient's condition and treatment in an actual medical setting and can be collected without burdening the patients, their families, or clinical staff, as they are accumulated from daily clinical practice [23]. Additionally, discussing and decision-making regarding patients’ values and goals of care is important for quality palliative care [24].

A QI set needs to be based on Donabedian’s theoretical framework, evaluating three aspects including structure, process, and outcome [25]. However, the QI sets developed to date have limitations. Most of them focus on process indicators and do not include all three aforementioned aspects. Second, their feasibility has not been tested during development [26]. Additionally, they are based on a consensus among project teams in the United States [26] and have not been considered for use in other countries, such as those in Asia. Since the roles and functions of ICUs vary according to the healthcare delivery system and culture of each country, it is imperative to develop QI sets for individual countries [27]. Ideally, QI sets should be evidence based; however, evidence of palliative care practices in the ICU is currently limited. Therefore, the present study aimed to develop a QI set for palliative care in ICUs, based on a systematic review and modified Delphi method, which is a formalized process of consensus building by expert groups, and to test its feasibility by reviewing EMR data.

## Methods

### Study design

The study was performed in two phases, namely the development of QIs using the modified Delphi method and the measurement of quality of palliative care in ICUs by reviewing EMRs.

### Development of QIs using the modified Delphi method

The modified Delphi method is a survey technique that involves multiple rounds to reach a consensus and is effective in determining expert consensus even when there is little or no conclusive evidence [28, 29]. The methodology and reporting of the modified Delphi study were based on Recommendations for the Conducting and Reporting of Delphi Studies [30].

**Fig. 1:**
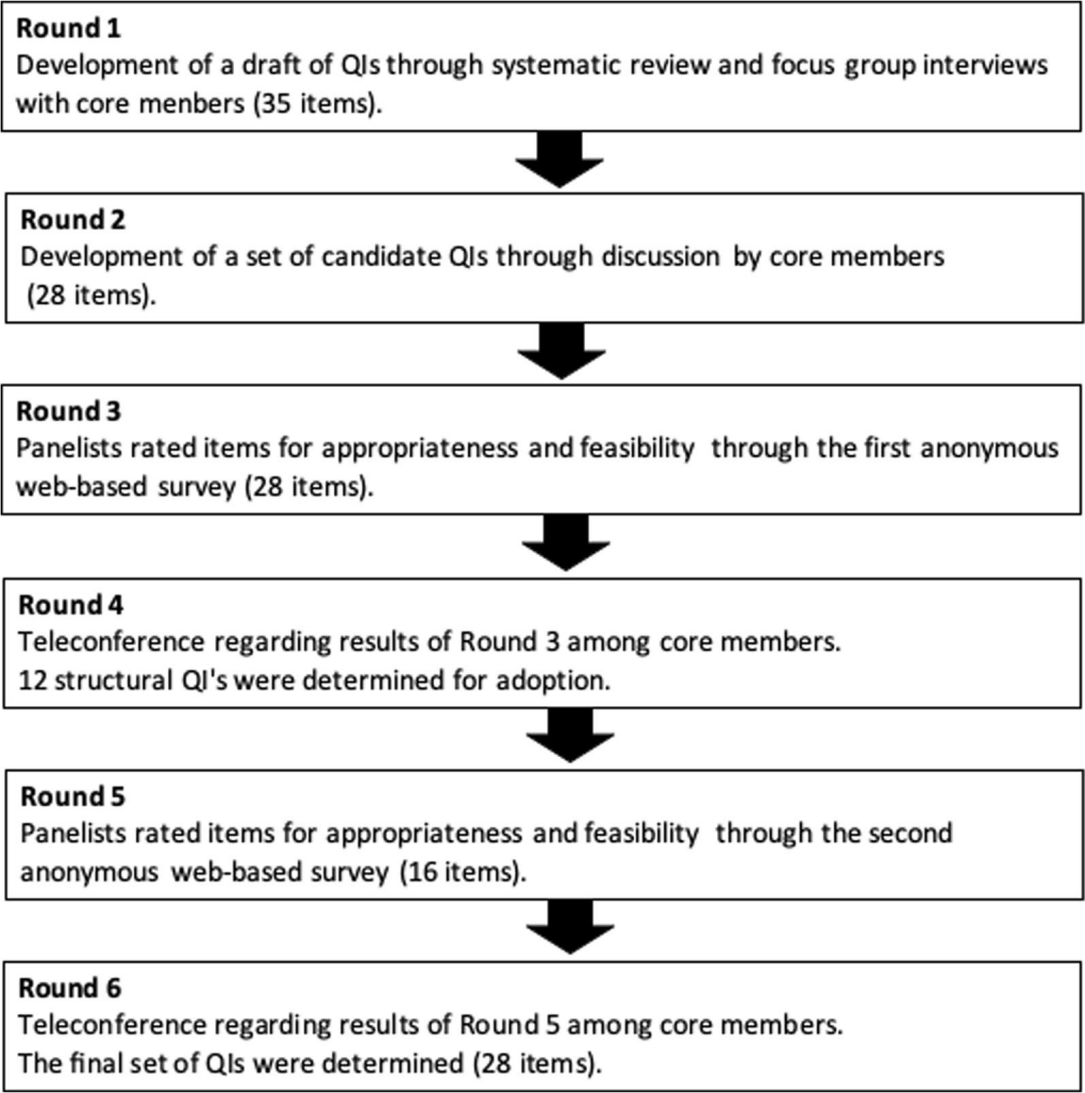
Modified Delphi process. This figure outlines the Delphi process conducted between December 2021 and July 2023

Figure 1 outlines the Delphi process conducted between December 2021 and July 2023.

In round 1, a systematic review of the QIs of palliative care in ICUs was conducted [26], and focus group interviews were held with eight core study members (palliative care physicians, intensivists, emergency physicians, nurses, and an expert on Delphi methodology).

In round 2, results of the systematic review and focus group interviews were considered, and the National Consensus Project Clinical Practice Guidelines for Quality Palliative Care, 4th Edition [31] was referred to in order to identify the best practices for palliative care of ICU patients and develop candidate QIs based on Donabedian's structure-process-outcome framework [25].

Subsequent rounds (rounds 3–6) comprised questionnaire surveys among expert panels, teleconferences, and e-mail discussions among the core members. One core member (Y.T.) sent an e-mail inviting potential panel members to participate in the study. The panel consisted of 16 members (five physicians, eight nurses, one physical therapist, one pharmacist, and one medical social worker) specialized in palliative or intensive care. Additional file 1 presents the details of the survey's rating by the panel of experts.

### *Pilot test of QIs for palliative care in ICUs *via* EMR review*

The QIs for palliative care in ICUs, developed in this study, were used to measure the QIs by reviewing EMRs.

The two sites included in this study were a general ICU and an emergency ICU within the same university hospital. The general ICU is a semi-closed ICU system that is primarily intended for patients with acute illnesses, postoperative patients, and patients with chronic illnesses such as chronic respiratory disease, heart failure, and renal failure. The emergency ICU is a closed ICU system that is primarily intended for emergency patients, including those with trauma, stroke, and other conditions requiring immediate medical intervention. The general ICU had 18 beds, and the emergency ICU had 16 beds. Both ICUs have intensivists on duty 24/7. The hospital is the largest in the region, with approximately one million patient visits per year. The hospital's ICUs have been designated as formal training facilities for intensivists.

Eligible patients were defined as follows: (1) patients admitted to the ICU on an urgent basis, and who stayed in the ICU for 48 or more consecutive hours or (2) patients admitted after scheduled surgery, who required mechanical ventilation for more than 48 consecutive hours. These patient criteria were established through a Delphi process to develop the QIs. We reviewed the EMRs of patients admitted to the ICU between January 1, 2019, and June 30, 2019. Data were collected from core-member nurses who were not working in the ICU. To verify the inter-rater reliability, another nurse independently reviewed the QIs and checked agreement with the main reviewer. We randomly selected 10% of the patients from our sample for testing. Data collectors also monitored the time required to measure the QIs per patient.

Each process and outcome QI was assessed at the individual level over the duration of ICU care. Aggregate quality scores were calculated by dividing the number of times palliative care was provided (numerator) by the number of eligible events (denominator). Structural QIs were assessed by reviewing the site policies and interviewing the site staff. Data collected included patient demographic data, survival data, disease severity, and length of ICU stay. A QI measurement manual was developed and used (Additional file 2).

Ethical approval for this study was granted by the Institutional Review Board of Tohoku University (No. 2022-1-1023), and the study conformed to the principles outlined in the Declaration of Helsinki.

### Statistical analysis

Summary statistics were calculated, and frequency distributions of the data were stratified according to each ICU. Performance frequency was calculated for each QI value. Continuous variables are presented as mean and standard deviation (SD), if normally distributed, and as median and interquartile range, if not normally distributed. To assess inter-rater reliability, we calculated the Cohen's kappa coefficient and the mean of differences. The data were analyzed using JMP version 17 (SAS Institute, Cary, NC, USA) and R version 4.2.0 (R Foundation for Statistical Computing, Vienna, Austria).

## Results

### Development of QIs using the modified Delphi method

In round 1, a systematic review was conducted, and 109 QIs were extracted from 5 literature sources [20, 22, 32–34]. The results were combined with the opinions from the focus group interviews to develop a draft of 35 candidate QIs.

In round 2, the core members discussed the appropriateness of each QI as an indicator to assess high- or low-quality palliative care in the ICU and narrowed the list to 28 candidate QIs.

The expert panel consisted of 16 participants with 20.0 ± 7.2 years of experience. Response rate of the surveys (rounds 3 and 5) was 100%; none of the responses were missing.

The panel members rated each QI item in rounds 3 and 5 (Table 1). In round 3, there was no item with a median score below seven for appropriateness, but five items (items 3, 5, 7,8, and 13) were rated low for feasibility. The core members mainly discussed feasibility issues (round 4), revised the QI titles, and modified the denominator settings. In round 5, feasibility ratings improved for most items. However, the following two items had median scores below seven: item 5, assessment of the patient's psychological distress, and item 7, assessment of the patient's spiritual and cultural practices.

**Table 1:** Delphi ratings for the advocated quality indicators

		Round 3 (n = 16)				Round 5 (n = 16)			
		Validity		Feasibility		Validity		Feasibility	
Indicators		Median	Agree (%)*	Median	Agree (%)*	Median	Agree (%)*	Median	Agree (%)*
*Process*									
1	Regular pain assessment	8	81.3	7.5	75.0	8	87.5	8	87.5
2	Appropriate pain management	8	75.0	7	62.5	8	93.8	7	75.0
3	Reassessment of pain after treatment and/or management	8	81.3	6.5	50.0	8	87.5	8	81.3
4	Regular delirium assessment	8	75.0	8	81.3	8	87.5	8	87.5
5	Assessment of the patient's psychological distress	7	50.0	5	31.3	7	56.3	6	43.8
6	Assessment of public social support needs	8	81.3	7	68.8	7.5	93.8	7	81.3
7	Assessment of the patient's spiritual and cultural practices	7	56.3	5	25.0	7	68.8	5	37.5
8	Identification of the patient's advance directive and ACP for treatment	8	75.0	6	43.8	8	93.8	7	68.8
9	Conducting an interdisciplinary family conference on palliative care	7.5	81.3	7	68.8	8	93.8	8	81.3
10	Transmission of key information regarding palliative care following ICU transfer	8	81.3	7	56.3	8	93.8	7	62.5
11	Assessments of psychological distress of family members	8	87.5	7	68.8	8	93.8	7	81.3
12	Documentation of the medical process regarding end-of-life decisions	8	81.3	7	56.3	8	87.5	7.5	75.0
13	Modification of medical care for it to be in concordance with the goals of care for patients at the end of life	8	81.3	6	37.5	8	93.8	7	68.8
*Outcome*									
14	Patient pain-free in the last 24 h of life	8	75.0	8	75.0	8	93.8	7.5	81.3
15	Avoid performing CPR when the patient does not want	8	68.8	8	75.0	8	87.5	8	81.3
*Structure*									
16	Use of standardized pain measurement scales	9	100.0						
17	Use of standardized dyspnea measurement scales	9	93.8						
18	Use of standardized thirst measurement scales	8.5	100.0						
19	End-of-life-specific symptom management care protocols or order sets	9	100.0						
20	Access to a palliative care team when pain and other physical symptoms are difficult to control	9	100.0						
21	Access to a specialized psychiatric team in presence of delirium, anxiety, or other difficult-to-control psychiatric symptoms	9	100.0						
22	A policy that allows for flexible visitation opportunities in accordance with the family's wishes	9	93.8						
23	Rooms with privacy for discussions between healthcare providers and family members	9	87.5						
24	The system to provide mental health care to patients and their families	9	93.8						
25	Leaflet for family members, including information on orientation to the ICU environment and delirium care	9	93.8						
26	A "Critical Care Mediator for Inpatients" is in place	7	68.8			7	75.0		
27	Regular opportunities for ICU staff to reflect on their end-of-life care experiences to support their emotional well-being	9	93.8						
28	Access to palliative care specialists and other professionals to discuss ethical issues related to treatment	9	100.0						

^*^Agreement was defined as the percentage of panelists assigning nine-point Likert scale scores of 7, 8, or 9. ICU, intensive care unit; ACP, advance care planning; CPR, cardiopulmonary resuscitation;

Discussions among core members (round 6) resulted in opinions regarding two of the above indicators, such as "the low feasibility of measurement ratings may simply reflect the fact that they are not currently being documented." Another commented, "spiritual and cultural practices are important to understand what kind of person the patient is, and are necessary as a consideration for diversity in the future." Therefore, we agreed that the two indicators would be necessary to improve palliative care in our country in future and decided to adopt the two items: item 5, assessment of the patient's psychological distress, and item 7, assessment of the patient's spiritual and cultural practices. Consequently, we modified the first 28 candidate QIs, and the new 28-item QI set consisting of eight domains became the final version (Table 2).

**Table 2:** Set of 28 quality indicators for palliative care in ICU

Indicators		Numerator	Denominator
Process			
*Assessment and management of patient's distress and needs*			
1	Regular pain assessment	Number of 4-h periods during the part of the 24-h day that a patient is in the ICU for which pain is assessed and recorded using a quantitative rating scale	Total number of 4-h periods during the part of the 24-h day that the patient is in the ICU*
2	Appropriate pain management	Number of records of assessed pain that was treated/managed or reasons why it was not treated/managed	Total number of periods during an ICU stay, in which the patient was assessed as having mild or greater pain (NRS: 4 or greater, BPS: 6 or greater, or CPOT: 3 or greater)**
3	Reassessment of pain after treatment and/or management	Number of records of reassessment within at least 2 h of the treatment/management implemented, whether it was effective or not	Total number of events in which patients admitted to the ICU were treated and managed for pain
4	Regular delirium assessment	Number of 8-h periods during the part of the 24-h day that a patient is in the ICU, for which delirium was assessed and recorded using a quantitative rating scale	Total number of 8-h periods during the part of the 24-h day that the patient is in the ICU***
5	Assessment of the patient's psychological distress	Number of patients with records indicating that the patient's psychological distress was assessed	Total number of patients with a GCS of 15 for more than 48 consecutive hours during the ICU stay
6	Assessment of public social support needs	Number of patients with records indicating that the need for formal social support for the patient was assessed	Total number of patients in the ICU
7	Assessment of the patient's spiritual and cultural practices	Number of patients with records indicating that the patient's spiritual and cultural aspects were assessed	Total number of patients in the ICU
*Patient- and family-centered decision making*			
8	Identification of the patient's advance directive and ACP for treatment	Number of patients with records identifying the patient's advance directive for treatment and ACP	Total number of patients in the ICU
9	Conduct of an interdisciplinary family conference on palliative care	Number of patients with records indicating that a multidisciplinary conference on palliative care that included the patient or family member was held, and a record of what was discussed	Total number of ICU patients who could identify a family member or a corresponding friend
*Continuity of care*			
10	Transmission of key information regarding palliative care following ICU transfer	Number of patients transferred from the ICU with records indicating that information discussed in multidisciplinary conferences on palliative care was passed on to the post-transfer team of health care providers	Total number of patients who were transferred out of the ICU (ex: transferred to another ward or another medical facility) with records indicating that a multidisciplinary conference on palliative care was conducted
*Psychological support for the patient's family*			
11	Assessments of psychological distress of family members	Number of patients with records indicating that the patient's family’s psychological distress was assessed	Total number of ICU patients who could identify a family member or a corresponding friend (they have visited)
*End-of-life care*			
12	Documentation of the medical process regarding end-of-life decisions	Number of patients for whom there is a record of discussion by a multidisciplinary health care team consisting of several physicians, including the primary physician, and other healthcare providers, such as nurses, regarding the determination that the patient is at the end of life	Total number of patients determined to be at the end of life in the ICU
13	Modification of medical care for it to be in concordance with the goals of care for patients at the end of life	Number of patients for whom there is a record of a reviewed or changed order that matches the patient's goals of care after the patient was determined to be at the end of life	Total number of patients determined to be at the end of life in the ICU
Outcome			
*End-of-life care*			
14	Patient pain-free in the last 24 h of life	Number of patients assessed as having no apparent pain in the 24 h before death	Total number of patients who died in the ICU
15	Avoid performing CPR when the patient does not want	Number of patients for whom CPR was not requested by the patient in the last hour before death	Total number of patients who had a DNAR policy and died in the ICU
Indicators		Numerator	Denominator
*Structure*			
*Setup and availability of resources and care protocols*			
16	Use of standardized pain measurement scales	Presence of a policy in the ICU of using quantitative measures to assess pain	ICU
17	Use of standardized dyspnea measurement scales	Presence of a policy in the ICU of using quantitative measures to assess dyspnea	ICU
18	Use of standardized thirst measurement scales	Presence of a policy in the ICU of using quantitative measures to assess thirst	ICU
19	End-of-life-specific symptom management care protocols or order sets	Presence of care protocols or order sets in the ICU for end-of-life-specific symptom management	ICU
20	Availability of a palliative care team	Availability of a palliative care team when pain or other physical symptoms are difficult to control	ICU
21	Availability of a specialized psychiatric team	Availability of a specialized psychiatric team in presence of delirium, anxiety, or other difficult-to-control psychiatric symptoms	ICU
*Support system for patient's family*			
22	A flexible visitation policy	A policy that allows for flexible visitation opportunities in accordance with the family's wishes	ICU
23	Rooms with privacy for discussions between health care providers and family members	A room with privacy for discussion between healthcare providers and family members is available	ICU
24	The system to provide mental health care to patients and their families	Have a system to provide mental health care for patients and their families	ICU
25	Leaflet for family members, including information on orientation to the ICU environment and delirium care	Leaflet for family members, including information on orientation to the ICU environment and delirium care	ICU
26	The "Critical Care Mediator for Inpatients" is in place in the ICU	A "Critical Care Mediator for Inpatients" is in place in the ICU	ICU
*Support system for ICU staff*			
27	Regular opportunities for ICU staff to reflect on their end-of-life care experiences to support their emotional well-being	Regular opportunities for ICU staff to reflect on their end-of-life care experiences to support their emotional well-being	ICU
28	Access to palliative care specialists and other professionals to discuss ethical issues related to treatment	Access to palliative care specialists and other professionals to discuss ethical issues related to treatment	ICU

^*^Denominator is the number of 4-h patient-nurse intervals (maximum of six per day) during the intensive care unit stay

^**^Denominator is the number of 4-h patient-nurse intervals (maximum of six per day) during the intensive care unit stay in which pain was assessed

^***^Denominator is the number of 8-h patient-nurse intervals (maximum of three per day) during the intensive care unit stay

NRS: Numeric rating scale; BPS: Behavioral pain scale; CPOT: Critical-care pain observation tool; GCS: Glasgow Coma Scale; ACP: Advance care planning; CPR: Cardiopulmonary resuscitation; DNAR: Do not attempt resuscitation

### Pilot test of the QIs for palliative care in ICUs via EMR review

Pilot testing of the developed QI set confirmed that process and outcome indicators were measurable from EMR data, whereas structural indicators were measurable from a survey of facility policies. Inter-rater reliability of the assessments was evaluated using Cohen's kappa coefficient. Overall, for QIs 5–15, the obtained Cohen's kappa value was 0.92 (95% confidence interval [CI] 0.87–0.97), indicating substantial inter-rater agreement. Additional file 3 presents the inter-rater reliability for each QI. Measurements per patient took a mean time of 32.2 ± 16.8 min.

### Patient characteristics

In 2019, a total of 1697 patients were admitted to the ICU. Of these, 850 patients were admitted from January 1, 2019, to June 30, 2019, and 262 (30.1%) were eligible for measurement of the QIs. Of the eligible patients, the patients scheduled for surgeries were all admitted to the General ICU, which accounted for 23.1% of the total number. The mean age of the patients was 63.1 ± 17.6 years, and 42.4% were admitted to the ICU for surgery. The mean duration of ICU stay was 7.1 days (Table 3).

**Table 3:** Characteristics of ICU patients

	General ICU	Emergency ICU	Total
Patient characteristics	n = 121	n = 141	n = 262
Age, mean (SD)	62.4 (± 16.4)	63.8 (± 18.6)	63.1 (± 17.6)
Sex, n (%)			
Female	43 (35.5%)	47 (33.3%)	90 (34.4%)
Male	78 (64.5%)	94 (66.7%)	172 (65.6%)
Employment, n (%)			
Employed	37 (30.6%)	42 (29.8%)	79 (30.2%)
Reason for ICU admission, n (%)			
Surgical	63 (52.1%)	48 (34.0%)	111 (42.4%)
Other	58 (47.9%)	93 (66.0%)	151 (57.6%)
Primary ICU diagnosis, n (%)			
Acute heart failure	17 (14.0%)	3 (2.1%)	20 (%)
Acute myocardial infarction or cardiogenic shock	16 (13.2%)	10 (7.1%)	26 (%)
Aortic disease	36 (29.8%)	0 (0.0%)	36 (%)
Pneumonia or respiratory failure	10 (8.3%)	23 (16.3%)	33 (%)
Sepsis or septic shock	12 (9.9%)	14 (9.9%)	26 (%)
Acute exacerbation of chronic obstructive pulmonary disease	2 (1.7%)	0 (0.0%)	2 (%)
Organ transplant	10 (8.3%)	0 (0.0%)	10 (%)
Stroke or Intracranial hemorrhage	5 (4.1%)	37 (26.2%)	42 (%)
Traumatic injury or burns	1 (0.8%)	35 (24.8%)	36 (%)
Other	12 (9.9%)	19 (13.5%)	31 (%)
Comorbidities, n (%)			
Congestive heart failure	8 (6.6%)	2 (1.4%)	10 (%)
Chronic pulmonary disease	8 (6.6%)	7 (5.0%)	15 (%)
Chronic renal replacement therapy	3 (2.5%)	4 (2.8%)	7 (%)
Diabetes mellitus	22 (18.2%)	25 (17.7%)	47 (%)
Liver disease	5 (4.1%)	4 (2.8%)	9 (%)
Metastatic cancer	6 (5.0%)	2 (1.4%)	8 (%)
Other cancer	23 (19.0%)	14 (9.9%)	37 (%)
Neuromuscular disease or epilepsy	6 (5.0%)	8 (5.7%)	14 (%)
Dementia	1 (0.8%)	7 (5.0%)	8 (%)
Ventilator therapy performed, n (%)	94 (77.7%)	70 (49.6%)	164 (62.6%)
Duration of ventilator therapy > 48 h, n (%)	82 (67.8%)	60 (42.6%)	142 (54.2%)
Acute physiology and chronic health Evaluation II score, mean (SD)	18.3 (± 5.9)	16.1 (± 7.2)	17.1 (± 6.7)
ICU length of stay, day (median, IQR)	7.6 (5.0–14.0)	7.1 (4.0–14.7)	7.1 (4.6–14.3)
Hospital length of stay, day (median, IQR)	38.6 (25.5–65.7)	20.7 (9.0–36.6)	27.8 (14.9–48.4)
Vital status at ICU discharge, n (%)			
Alive	102 (84.3%)	118 (83.7%)	220 (84.0%)
Expired	19 (15.7%)	23 (16.3%)	42 (16.0%)
Vital status at hospital discharge, n (%)			
Alive	94 (77.7%)	114 (80.9%)	208 (79.4%)
Expired	27 (22.3%)	27 (19.1%)	54 (20.6%)

ICU: intensive care unit; SD: standard deviation; IQR: interquartile range

### Structures, processes, and outcomes of palliative care in ICUs

Table 4 presents the frequencies of process performance and outcome indicators for the entire study sample. Of the 15 process and outcome indicators, 7 with performance frequencies less than 50% were as follows: (1) regular pain assessment, (3) reassessment of pain after treatment and/or management, (5) assessment of patient’s psychological distress, (7) assessment of patient’s spiritual and cultural practices, (8) identification of the patient’s advance directive and advance care planning (ACP) for treatment, (9) conducting an interdisciplinary family conference on palliative care, and (11) assessment of family members’ psychological distress. Additionally, there were some differences in the percentage of practices across the sites. The largest differences between the sites were regarding (1) regular pain assessment and (14) pain-free status in the last 24 h of life. “Patient pain-free in last 24 h of life” could not be evaluated due to the low implementation rate of “regular pain assessment” in emergency ICU.

**Table 4:** Frequency of performance of the measures of processes and outcomes of palliative care in ICU

		General ICU (n = 121)			Emergency ICU (n = 141)			Total (n = 262)		
	Indicators	Numerator (n)	Denominator (n)	%	Numerator (n)	Denominator (n)	%	Numerator (n)	Denominator (n)	%
Process										
1	Regular pain assessment*	5405	9282	58.2%	2818	8544	33.0%	8223	17826	46.1%
2	Appropriate pain management^†^	142	182	78.0%	125	177	70.6%	267	359	74.4%
3	Reassessment of pain after treatment and/or management	73	142	51.4%	41	125	32.8%	114	267	42.7%
4	Regular delirium assessment^‡^	3355	4524	74.2%	3379	4218	80.1%	6734	8742	77.0%
5	Assessment of the patient's psychological distress	40	86	46.5%	23	53	43.4%	63	139	45.3%
6	Assessment of social support needs	110	121	90.9%	126	141	89.4%	236	262	90.1%
7	Assessment of the patient's spiritual and cultural practices	7	121	5.8%	6	141	4.3%	13	262	5.0%
8	Identification of the patient's advance directive and ACP for treatment	12	121	9.9%	14	141	9.9%	26	262	9.9%
9	Conduct of an interdisciplinary family conference on palliative care	34	116	29.3%	42	139	30.2%	76	255	29.8%
10	Transmission of key information regarding palliative care following ICU transfer	14	23	60.9%	18	23	78.3%	32	46	69.6%
11	Assessments of psychological distress of family members	49	116	42.2%	59	139	42.4%	108	255	42.4%
12	Documentation of the medical process regarding end-of-life decisions	20	25	80.0%	34	35	97.1%	54	60	90.0%
13	Modification of medical care for it to be in concordance with the goals of care for patients at the end of life	23	25	92.0%	35	35	100.0%	58	60	96.7%
Outcome										
14	Patient pain-free in the last 24 h of life	19	19	100.0%	10	23	43.5%	29	42	69.0%
15	Avoid performing CPR when the patient does not want	18	18	100.0%	22	23	95.7%	40	41	97.6%

*Denominator is the number of 4-h patient-nurse intervals (maximum of six per day) during the intensive care unit stay

^†^Denominator is the number of 4-h patient-nurse intervals (maximum of six per day) during the intensive care unit stay in which pain was assessed

^‡^Denominator is the number of 8-h patient-nurse intervals (maximum of three per day) during the intensive care unit stay

Table 5 presents the results of evaluation of the structural QIs of the two ICUs. The results were similar since the ICUs were located within the same hospital.

**Table 5:** Performance frequency of measures of palliative care structures in the ICU

		Total (n = 2)*
	Indicators	%
Structure		
16	Use of standardized pain measurement scales	100.0%
17	Use of standardized dyspnea measurement scales	0.0%
18	Use of standardized thirst measurement scales	0.0%
19	End-of-life-specific symptom management care protocols or order sets	0.0%
20	Access to a palliative care team when pain and other physical symptoms are difficult to control	100.0%
21	Access to a specialized psychiatric team in presence of delirium, anxiety, or other difficult-to-control psychiatric symptoms	100.0%
22	A policy that allows for flexible visitation opportunities in accordance with the family's wishes	100.0%
23	Rooms with privacy for discussions between health care providers and family members	100.0%
24	A system to provide mental health care to patients and their families	100.0%
25	Leaflet for family members, including information on orientation to the ICU environment and delirium care	100.0%
26	A "Critical Care Mediator for Inpatients" is in place	0.0%
27	Regular opportunities for ICU staff to reflect on their end-of-life care experiences to support their emotional well-being	100.0%
28	Access to palliative care specialists and other professionals to discuss ethical issues related to treatment	100.0%

^*^Structure QI is a per-site evaluation; thus, the total represents two sites, the General ICU and Emergency ICU

ICU: intensive care unit

## Discussion

In this study, based on a systematic review and expert consensus, we developed a QI set for palliative care in ICUs that consisted of 28 items across eight domains. Pilot testing demonstrated the set of QIs to be feasible and measurable. Additionally, the pilot test results suggested several potential improvements in palliative care in ICU settings.

This study developed a QI set that followed Donabedian’s structure-process-outcome framework. Additionally, the QI set was based on a systematic review and covered eight domains of the Clinical Practice Guidelines for High-Quality Palliative Care, which provide a foundation for improving the quality and delivery of palliative care in the United States [31]. In the Delphi rounds of this study, two items, namely “assessment of the patient's psychological distress” and “assessment of the patient's spiritual and cultural practices,” were rated as having low feasibility. Low-feasibility indicators are often not documented in EMRs, and hence, the potential need for quality improvement is missed [35, 36]. We included these two items in the QI set, assuming that low feasibility due to non-documentation is indicative of poor quality of care.

This study confirmed the feasibility and inter-rater reliability of QI measurements using medical record data from two ICUs. An average time of 32 min was spent on each patient to measure the process outcome QIs. Since this was a pilot study, the time taken for these measurements may reduce as people become accustomed to the task of measurement. A study testing the quality-of-care indicators for patients with cancer reported that the review of 92 indicators from EMRs takes approximately 2.4 h per patient [37, 38]. Thus, the necessary information may be collected within a reasonable time, but the simplicity and feasibility of the evaluation method should be further enhanced to promote quality palliative care worldwide. In this set of QIs, the evaluator spends more than half of the time on the analysis of the patient's assessment of pain and delirium. In this regard, to reduce the evaluation time, data covering only 48 h after admission to the ICU could be assessed. In addition, natural language processing and machine learning techniques have been demonstrated to handle textual data from EMRs with little burden on healthcare providers or patients in palliative care settings [39–41]. This state-of-the-art technology can enable rapid review and feedback of documentation. Therefore, the simplification of QI measurement methods and the use of technology in evaluation are important to increase the feasibility of continuous evaluation.

Pilot testing of the medical record review survey identified the following four aspects that required improvement: assessment of regular pain, identification of the patient's advance directive and ACP for treatment, conducting an interdisciplinary family conference on palliative care, and assessment of the psychological distress experienced by family members.

In this study, the frequency of pain assessment was 46%. Previous studies conducted in the United States reported pain assessment frequencies of 76–87% [20, 21], hence, suggesting the need for improved pain assessment in Japan. The outcome indicator “no pain in the 24 h before death” was 69% in this study, which is not greatly different from 72% in the United States [34]. However, because the frequency of regular pain assessment was low, the presence or absence of pain may not have been accurately assessed, thereby possibly affecting the outcomes. The guidelines emphasize that the presence of pain and need for pain management should not be dismissed in critically ill patients, who are often unable to communicate clearly [42, 43]. Therefore, continuous assessment and recording of pain severity would ensure that pain presence is not neglected in critically ill patients; this would improve the quality of ICU care, including the efficient use of analgesics and sedative medications.

In this survey, approximately 10% of respondents confirmed their advance directive and ACP for treatment, and approximately 30% discussed the patient's quality of life and values at a multidisciplinary conference that included family members. This was similar to the results of a survey of three ICUs in the United States, which reported advanced directive status (31%) and conduction of family conferences (19%) [21]. In a national institutional survey of ICUs in Japan regarding the frequency of multidisciplinary conferences on palliative care, 75% of ICUs reported that conferences were never held, and only 8% responded that they were held four or more times a month [44]. Interventions in family conferences and communication are necessary to provide patient-centered care and increase family satisfaction and trust in healthcare providers [45]. In recent years, the decision-making process for treatment in ICUs has recommended the implementation of discussions with patients and their families about goals of care within 5–7 days of ICU admission and holding of weekly multidisciplinary conferences [46]. Confirming ACPs regarding treatment and conducting family conferences are important for achieving treatment goals that are in line with the patient's wishes, that is, to provide patient-centered care.

A unique element of this QI set is the inclusion of a QI that assessed families’ psychological distress. In ICUs, where patients often lack decision-making capacity or are unable to express their treatment preferences, the patient's family is an important component of palliative care. Patients’ families, who are often surrogate decision-makers in the unique environment of the ICU, also experience psychological distress, since they are in crisis [9, 10]. Therefore, family members of ICU patients should be recognized as beneficiaries of palliative care, and assessment of family members' psychological distress is considered important.

The structure indicator in this QI set consists of three domains, namely "Setup and availability of resources and care protocols," "Support system for patient's family," and "Support system for ICU staff." Support for ICU staff is a distinctive domain. Prevention of burnout through a support system of ICU staff is an important indicator that leads to the continuous provision of high-quality care to ICU patients and their families [47]. This survey could not evaluate the structure of palliative care in ICUs in Japan, since data were obtained from only two ICUs. In future, surveys should be conducted at more facilities to clarify the current status at the national level.

### Implications for future

This study developed a QI set comprising QIs that can be measured using data extracted from medical records, allowing easy measurability and sustained and continued use of the QIs, with limited measurement of patient outcomes. Future studies are warranted that evaluate patient-family experience outcomes from questionnaires regarding patient health [48] or assess family satisfaction using the questionnaires regarding intensive care units [49] in conjunction with measurement of this QI set. To improve the quality of ICUs, establishment of a system that, in parallel with educational interventions for staff, feeds the results of quality measurement surveys back to clinical practice in a cycle of improvement, would be required [50]. Kruser et al. conducted a large study that enrolled 68 ICUs in the United States and evaluated the three aspects of structure, process, and outcome to identify unit-level variation. To improve quality of palliative care in ICUs in future, they proposed analyzing the potential characteristics of units that provide high-quality care [34]. Even in Japan, where palliative care in ICUs is still developing, a multicenter survey needs to be conducted and benchmarked to assess the quality of care at the national level. Continuous research utilizing this QI set needs to be conducted to monitor the cycle of change and improvement in clinical practice, owing to the implementation of awareness and educational interventions.

### Study limitations

This study had several limitations. First, since the measures in this QI set were based on medical record data, we may have underestimated the actual care provided in the absence of documentation. On the other hand, there are concerns about the risk of the possibility that the measures are not actually being provided even though they are being documented in the EMR and the risk of copy and paste duplication of documents [23]. However, we believe that documentation of pain assessment and discussion of goals of care are important in good-quality palliative care and may be quality indicators in and of itself. Second, since the study was conducted in two units within a single hospital, generalization is limited. Different hospital sizes and regions may have different demographic characteristics and disease coverage. In addition, the way in which medical records are written can vary depending on the hospital's situation and organizational culture. To deal with the issue of representativeness of the pilot test, the study was set up in a large-sized university hospital that serves as a teaching facility for intensive care physicians, and thus requires a certain level of medical care. Furthermore, by validating the study in two different types of units, we endeavored to accommodate variations. As a logical next step in the research, a multicenter study is being considered for further validation of our findings. Finally, although we developed a comprehensive QI set related to patient/family-centered and ICU staff-support domains, evidence associating most structures and processes of palliative care in the ICU with outcomes remains limited [51]. This would need to be modified in future to adjust to the changes in practice and accumulate new evidence.

## Conclusions

A 28-item QI set was developed using the modified Delphi method and measured using EMR data, thereby providing a tool for assessment of the quality of palliative care in ICUs. Pilot testing using medical record data from two ICUs confirmed its feasibility and measurability. Our pilot study suggested the aspects of palliative care in ICUs in Japan that need to be improved, further highlighting the importance of conducting a nation-wide multicenter survey. To conduct large-scale surveys in future, we need to test the QI set further, with focus on decreasing the burden of measurement.

### Supplementary Information

**Additional file 1. **The modified Delphi method.

**Additional file 2. **QI measurement manual.

**Additional file 3. **Table. Inter-rater reliability verification.

## Data Availability

The data sets used and analyzed during the current study are available from the corresponding author upon reasonable request.

## Abbreviations

ICU: Intensive care unit
QI: Quality indicator
EMR: Electronic medical record
ACP: Advance care planning
SD: Standard deviation
CI: Confidence interval
NRS: Numeric rating scale
BPS: Behavioral pain scale
CPOT: Critical-care pain observation tool
GCS: Glasgow coma scale
CPR: Cardiopulmonary resuscitation
DNAR: Do not attempt resuscitation
IQR: Interquartile range
