# Supplementary material for: Development of quality indicators for palliative care in intensive care units and pilot testing them via electronic medical record review
Source: J Intensive Care. 2024 Jan 9;12:1. doi: 10.1186/s40560-023-00713-z (PMC10775577; doi:10.1186/s40560-023-00713-z)
Supplement: Supplementary file 1 — Additional file 1. The modified Delphi method. [file 40560_2023_713_MOESM1_ESM.docx]

**Additional file 1. The modified Delphi method**

We developed a questionnaire and worksheet for each of the candidate quality indicators (QIs) with brief titles, denominator and numerator definitions, data sources, and available evidence. Panelists rated each QI using a 9-point Likert scale for the appropriateness of the quality of palliative care in intensive care units (1–3: inappropriate, 4–6: intermediate, and 7–9: appropriate) and feasibility of extraction via electronic medical records (1–3: infeasible, 4–6: intermediate, and 7–9: feasible). In addition, a free-text section was provided to solicit opinions on each QI. We set the criteria for adopting a QI as follows: each QI's appropriateness and feasibility score must be in agreement; specifically, there must be no more than three ratings in the "1–3" (inappropriate/infeasible) range, and 70% of the responses must converge to the "7–9" (adequate/feasible) range, including the median. Items that were not agreed upon in the panel survey were discussed and finalized by the core members. The panelists' response data were collected anonymously using Google Forms and compiled into a worksheet. This approach was adopted owing to the logistical difficulties in gathering all members at the same time and at the same place.

In round 5, a second panel survey included a supplemental document containing the overall results of the first survey and responses to the opinions expressed in the open-ended sections of the first survey. Thus, each panelist could refer to the overall responses and their previous responses.
