# Supplementary material for: Development of quality indicators for palliative care in intensive care units and pilot testing them via electronic medical record review
Source: J Intensive Care. 2024 Jan 9;12:1. doi: 10.1186/s40560-023-00713-z (PMC10775577; doi:10.1186/s40560-023-00713-z)
Supplement: Supplementary file 2 — Additional file 2. QI measurement manual. [file 40560_2023_713_MOESM2_ESM.docx]

Additional file 2. QI measurement manual

June 12, 2023

**Quality Indicators for Palliative Care in Intensive care units (ICUs):**

**Measurement using electronic medical records**

This indicator has a total of 28 items. Process Indicators (13 items) and Outcome Indicators (2 items) assess the quality of care at the patient level. Structure indicators (13 items) assess the quality of care at the institutional level.

**The definition of "patient admitted to the ICU" as a target for measurement of this indicator is as follows.**

**(1) Unscheduled admissions include patients who stayed in the ICU for more than 48 consecutive hours.**

**or**

**(2) Patients admitted after scheduled surgery who required ventilation therapy for more than 48 consecutive hours.**

**Process Indicators**

1. Regular pain assessment
2. Appropriate pain management
3. Reassessment of pain after treatment and/or management
4. Regular delirium assessment
5. Assessment of the patient's psychological distress
6. Assessment of public social support needs
7. Assessment of the patient's spiritual and cultural aspects
8. Identification of the patient's advance directive and ACP for treatment
9. Conduct of an interdisciplinary family conference on palliative care
10. Transmission of key information regarding palliative care following ICU transfer
11. Assessments of psychological distress of family members
12. Documentation of the medical process regarding end-of-life decisions
13. Modification of medical care for it to be in concordance with the goals of care for patients at the end of life

**Outcome Indicators**

1. Patient pain-free in last 24 h of life
2. Avoid performing CPR when the patient does not want

**Structure indicators**

1. Use of standardized pain measurement scales
2. Use of standardized dyspnea measurement scales
3. Use of standardized thirst measurement scales
4. End-of-life specific symptom management care protocols or order sets
5. Availability of a palliative care team
6. Availability of a specialized psychiatric team
7. A flexible visitation policy
8. Rooms with privacy for discussions between healthcare providers and family members
9. The system to provide mental health care to patients and their families
10. Leaflet for family members, including information on orientation to the ICU environment and delirium care
11. A "Critical Care Mediator for Inpatients" is in place
12. Regular opportunities for ICU staff to reflect on their end-of-life care experiences to support their emotional well-being
13. Access to palliative care specialists and other professionals to discuss ethical issues related to treatment

〈Measurement Methods in Detail〉

**Process Indicators**

1. **Regular pain assessment**

Indicator Definition: Documentation of pain assessment performed using quantitative rating scales such as BPS, CPOT, NRS.

Numerator: Number of 4-h periods during the part of the 24-h day that a patient is in the ICU for which pain is assessed and recorded using a quantitative rating scale.

- If the patient can self-report, use NRS or NRS Visual.
- If self-reporting is not possible, use BPS or CPOT, which are behavioral assessment-type tools.
- A range of 1 h before and after is acceptable for the timing of the 4-hourly recordings. (Even if the number of evaluations in a 24-hour period in the numerator exceeds 6, the number of evaluations shall be 6 = 100%.)
- Set a standard daily start time for each facility, taking into account the nurse's work system and recording time (e.g. record at 6:00 a.m. as a starting point, divided into 4-h periods).

Denominator: Total number of 4-h periods that a patient is in the ICU during the part of the 24-h day that the patient is in the ICU.

- If the period of measurement was 24 h, the denominator would be 6.

Intended sample: ICU patients who meet the definition of target eligibility criteria.

Potential exclusions: Time spent off the unit and no longer in the care of the ICU nurse (e.g., scan, in operating room, etc.).

Data source and collection methods: Electronic medical record data. All records of the eligible patients during the ICU stay. (Physician records, nurse records, progress notes, etc.)

1. **Appropriate pain management**

Indicator Definition: Documentation of "treatment and/or management" for patients with pain.

Numerator: Number of records of assessed pain that was treated/managed or reasons why it was not treated/managed.

- Patients with values of NRS: 4 or higher, or BPS: 6 or higher, or CPOT: 3 or higher are defined as patients with pain.
- "Treatment and/or management" include pharmacologic interventions, such as increased or rapid dosing of analgesics, or nonpharmacologic interventions, such as positioning and massage.
- Documentation of the reason why it was not performed is assumed to be "temporary worsening of pain due to patient's motion is considered, so we will observe the patient at rest", etc.

Denominator: Total number of periods during the ICU stay in which the patient was assessed as having mild or greater pain (NRS: 4 or greater, or BPS: 6 or greater, or CPOT: 3 or greater).

Intended sample: ICU patients who meet the definition of target eligibility criteria.

Potential exclusions: Time spent off the unit and no longer in the care of the ICU nurse (e.g., scan, in operating room, etc.).

Data source and collection methods: Electronic medical record data. All records of the eligible patients during the ICU stay. (Physician records, nurse records, progress notes, etc.)

1. **Reassessment of pain after treatment and/or management**

Indicator Definition: Documentation of reassessment of pain after treatment and/or management.

Numerator: Number of records of reassessment within at least 2 h of the treatment and/or management implemented, whether it was effective or not.

- "Treatment and/or management" include pharmacologic interventions, such as increased or rapid dosing of analgesics, or nonpharmacologic interventions, such as positioning and massage.
- Similar to the prior implementation assessment, reassess using a quantitative rating scales such as BPS, CPOT, NRS.
- Record of reassessment is considered an achievement, even if the pain has not improved.
- If the timing overlaps with the recording of the regular 4-hourly assessment, it is accepted as a record of the reassessment.

Denominator: Total number of events in which patients admitted to the ICU were treated and/or managed for pain.

Intended sample: ICU patients who meet the definition of target eligibility criteria.

Potential exclusions: Time spent off the unit and no longer in the care of the ICU nurse (e.g., scan, in operating room, etc.).

Data source and collection methods: Electronic medical record data. All records of the eligible patients during the ICU stay. (Physician records, nurse records, progress notes, etc.)

1. **Regular delirium assessment**

Indicator Definition: Documentation of delirium assessment performed using quantitative rating scales.

Numerator: Number of 8-h periods during the part of the 24-h day that a patient is in the ICU for which delirium is assessed and recorded using a quantitative rating scale.

- A range of 1 h before and after is acceptable for the timing of the 8-hourly recordings. (Even if the number of evaluations in a 24-h period in the numerator exceeds 3, the number of evaluations shall be 3 = 100%.)
- Patients under deep sedation or severely impaired consciousness who are unable to respond to a call (e.g., RASS-4 to -5) for a period of time in which delirium is difficult to assess shall be recorded as either "unable to assess" or "RASS-4 to -5".

Denominator: Total number of 8-h periods that a patient is in the ICU during the part of the 24-h day that the patient is in the ICU.

- If the period of measurement was 24 h, the denominator would be 3.

Intended sample: ICU patients who meet the definition of target eligibility criteria.

Potential exclusions:

- - Time spent off the unit and no longer in the care of the ICU nurse (e.g., scan, in operating room, etc.).
  - If the patient is evaluable for delirium and has not had a change in mental status in the past 24 h or more, exclude that period from the denominator. (If there is a change in mental status after the exclusion, include the period from that point in the denominator.)

Data source and collection methods: Electronic medical record data. All records of the eligible patients during the ICU stay. (Physician records, nurse records, progress notes, etc.)

1. **Assessment of the patient's psychological distress**

Indicator Definition: Documentation of assessment of the patient's psychological distress.

Numerator: Number of patients with records indicating that the patient's psychological distress was assessed.

- Assessments of the patient's psychological aspects are described, including restlessness, fear, self-doubt, mood blocking, sleep disturbances, and feelings of dreariness, identifying coping methods and a supportive presence.
- At least one assessment and documentation of the assessment during the period of ICU admission.
- Even if there are no psychological problems identified during the assessment, describe the findings.

Denominator: Total number of patients with a GCS of 15 for more than 48 consecutive hours during the ICU stay.

- We formulated the denominator because we thought that psychological distress should be assessed in patients who have a clear consciousness as a minimal requirement.

Intended sample: ICU patients who meet the definition of target eligibility criteria.

Potential exclusions:

- - Exclude patients with impaired consciousness.

Data source and collection methods: Electronic medical record data. All records of the eligible patients during the ICU stay. (Physician records, nurse records, progress notes, etc.)

1. **Assessment of public social support needs**

Indicator Definition: Documentation of assessment of social support needs.

Numerator: Number of patients with records indicating that the need for formal social support for the patient was assessed.

- Records such as documents ordering social workers to offer intervention, including records of consultation with the patients or documents from the family members to the social workers. (Creation of "discharge support plan" for the hospitalization/discharge support addition, family interviews with social workers, and discharge support conferences held by medical personnel).
- At least one assessment and documentation of the assessment during the period of ICU admission.

Denominator: Total number of patients in the ICU.

Intended sample: ICU patients who meet the definition of target eligibility criteria.

Data source and collection methods: Electronic medical record data. All records of the eligible patients during the ICU stay. (Physician records, nurse records, progress notes, etc.)

1. **Assessment of the patient's spiritual and cultural aspects**

Indicator Definition: Documentation of assessment of the patient's spiritual and cultural aspects.

Numerator: Number of patients with records indicating that the patient's spiritual and cultural aspects were assessed.

- Assessment from a spiritual care perspective. *
- Documentation of assessment of the patient's spiritual aspects, including loss of role, loss of sense of physical control, remnants of mind, fear of death, and identification of religious beliefs and behaviors.
- At least one assessment and documentation of the assessment during the period of ICU admission.

*Refer to “SpiPas” core concepts, definitions, sample questions, and examples of specific expressions. (*Ichihara K, et al. Palliat Support Care. 2019*)

Denominator: Total number of patients in the ICU.

Intended sample: ICU patients who meet the definition of target eligibility criteria.

Data source and collection methods: Electronic medical record data. All records of the eligible patients during the ICU stay. (Physician records, nurse records, progress notes, etc.)

1. **Identification of the patient's advance directive and ACP for treatment**

Indicator Definition: Documentation of the patient's advance directives regarding treatment and confirmation of the content of the ACP.

Numerator: Number of patients with records identifying the patient's advance directive for treatment and ACP.

- Documentation confirming the patient's advance directive and ACP for treatment with the patient, family, or health care provider (attending physician, ward nurse) who has taken over patient care in the ICU.
- "Patient's advance directive and ACP for treatment" includes not only the patient's written intention, such as advance directives, but also the patient's daily behaviors and actions regarding values and views on life and death, and information about the patient's expectations from the family.
- A record of DNAR (Do not Attempt Resuscitation) signed after ICU admission is not sufficient.

Denominator: Total number of patients in the ICU.

Intended sample: ICU patients who meet the definition of target eligibility criteria.

Data source and collection methods: Electronic medical record data. All records of the eligible patients during the ICU stay. (Physician records, nurse records, progress notes, etc.)

1. **Conduct of an interdisciplinary family conference on palliative care**

Indicator Definition: Documentation of the contents of the multidisciplinary meetings that include the patient or the patient’s family on palliative care.

Numerator: Number of patients for whom a multidisciplinary conference on palliative care that included the patient or a family member was held, with a record of what was discussed.

- ”Interdisciplinary" includes the attending physician (either the primary physician or the ICU physician), nurses, social workers, and psychologists, with nurses participating to the extent possible.
- The minimum number of participants should be at least two healthcare providers, including the attending physician and a nurse.
- ”The "Palliative Care Conference" include discussions on the following four topics *
  1. The patient's condition (diagnosis and prognosis)
  2. The patient's needs and preferences (including the family's estimation of the patient's needs and preferences) and quality of life
  3. Surrounding circumstances (family opinions, level of burden, social support, hospital policies)
  4. The treatment plan and goals of care

* All topics (1) ~ (4) must be discussed and documented during the period of ICU admission. Discussions may be divided into multiple sessions to ensure that all the four topics are discussed

* At least one of these meetings should be attended by the patient or by a family member or friend.

Denominator: Total number of ICU patients who could identify a family member or a corresponding friend.

Intended sample: ICU patients who meet the definition of target eligibility criteria.

Data source and collection methods: Electronic medical record data. All records of the eligible patients during the ICU stay. (Physician records, nurse records, progress notes, etc.)

1. **Transmission of key information regarding palliative care following ICU transfer**

Indicator Definition: Existence of documentation that the contents of the multidisciplinary conferences on palliative care after transfer from the ICU are passed on to the team of healthcare providers.

Numerator: Number of patients transferred from the ICU with records indicating that the results of the multidisciplinary conferences on their palliative care were passed on to the post-transfer team of healthcare providers.

- The "record of interdisciplinary conference on palliative care" must include information regarding the following aspects of the period of ICU admission.

1. The patient's condition (diagnosis and prognosis)
2. The patient's needs and preferences (including the family's estimation of the patient's needs and preferences) and quality of life
3. Surrounding circumstances (family opinions, level of burden, social support, hospital policies)
4. The treatment plan and goals of care

- An attached document showing the contents of the conference as "See Conference Record" is considered an accomplishment.

Denominator: Total number of patients who were transferred out of the ICU (ex: transferred to another ward or another medical facility) with records indicating that an interdisciplinary conference on palliative care was conducted.

Intended sample: ICU patients who meet the definition of target eligibility criteria.

Data source and collection methods: Electronic medical record data. All records of the eligible patients during the ICU stay. (Physician records, nurse records, progress notes, etc.)

1. **Assessments of psychological distress of family members**

Indicator Definition: Documentation of assessment of the psychological distress of the patients’ families.

Numerator: Number of patients with records indicating that the psychological distress of their families was assessed.

- Assessments of the psychological characteristics of the patient’s families are described, including restlessness, fear, self-doubt, mood blocking, sleep disturbances, and feelings of dreariness, identifying coping methods and a supportive presence.
- At least one assessment and documentation of the assessment during the period of ICU admission.
- Even if there are no psychological problems identified during the assessment, describe the findings.

Denominator: Total number of ICU patients who could identify a family member or a corresponding friend (they have visited).

Intended sample: ICU patients who meet the definition of target eligibility criteria.

Data source and collection methods: Electronic medical record data. All records of the eligible patients during the ICU stay. (Physician records, nurse records, progress notes, etc.)

1. **Documentation of the medical process regarding end-of-life decisions**

Indicator Definition: Records indicating that the end-of-life decisions were discussed by a multidisciplinary team comprising several physicians (preferably from several departments), including the primary physician, and nurses.

Numerator: Number of patients for whom there is a record of discussion by a multidisciplinary health care team comprising several physicians, including the primary physician, and other healthcare providers, such as nurses, regarding the patient being in a terminal condition.

- Definition of end-of-life for this indicator is as follows.

"The end of life in intensive care refers to the phase when patients are evaluated as no longer expected to survive even if appropriate treatment is given to them in ICUs. It is determined by the medical team, taking into consideration not only the medical aspects but also the state and values of the individual patient."

- It is important that the decision that an ICU patient is terminally ill and the subsequent responses to this decision are taken by a medical team, comprising several physicians (preferably from several departments), including the attending physician, and nurses, as a consensus.

Denominator: Total number of patients determined to be at the end of life in the ICU.

Intended sample: ICU patients who meet the definition of target eligibility criteria.

Data source and collection methods: Electronic medical record data. All records of the eligible patient during the ICU stay. (Physician records, nurse records, progress notes, etc.)

1. **Modification of medical care for it to be in concordance with the goals of care for patients at the end of life**

Indicator Definition: Measure whether goals of care are being modified and orders are being changed with the goal of reducing patient suffering and preserving dignity.

Numerator: Number of patients for whom there is a record of a reviewed or changed order that matches the patient's goals of care after the patient was determined to be at the end of life.

- Definition of end-of-life for this indicator is as follows.

"The end of life in intensive care refers to the phase when patients are evaluated as no longer expected to survive even if appropriate treatment is given to them in ICUs. It is determined by the medical team, taking into consideration not only the medical aspects but also the state and values of the individual patient."

- Documentation of any review or modification of goals of care (e.g., Comfort Measures Only, etc.).
- Modification of medical orders refers to changes made with a focus on whether they meet the patient's goals of care, reduce suffering, or do not compromise the patient's dignity.

Denominator: Total number of patients determined to be at the end of life in the ICU.

Intended sample: ICU patients who meet the definition of target eligibility criteria.

Data source and collection methods: Electronic medical record data. All records of the eligible patients during the ICU stay. (Physician records, nurse records, progress notes, etc.)

**Outcome Indicators**

1. **Patient pain-free in last 24 h of life**

Indicator Definition: Percentage of patients assessed as having no apparent pain in the 24 h before death.

Numerator: Number of patients assessed as having no pain in the 24 h before death. (As assessed by BPS or CPOT).

- "No pain" is defined as BPS: 5 or less or CPOT: 2 or less.

Denominator: Total number of patients died in the ICU.

Intended sample: ICU patients who meet the definition of target eligibility criteria.

Data source and collection methods: Electronic medical record data. All records of the eligible patients during the ICU stay. (Physician records, nurse records, progress notes, etc.)

1. **Avoid performing CPR when the patient does not want**

Indicator Definition: Percentage of patients who were not provided cardiopulmonary resuscitation in the hour before death according to the patients’ preferences.

Numerator: Number of patients who declined CPR in last hour before death.

- This indicator measures compliance with the DNAR policy as determined through discussions between the patient or a family member who is aware of the patient's wishes and the medical team.
- If the patient is unable to express his/her wish, the best policy is determined through discussions between the patient's family and the medical team and is presumed to be the patient's own wish.
- In-hospital deaths include ward deaths within 7 days of ICU discharge. It is intended to assess whether the DNAR policy was correctly communicated and adhered to after ICU transfer.

Denominator: Total number of patients who signed up for DNAR policy and died in the ICU or in the hospital.

Intended sample: ICU patients who meet the definition of target eligibility criteria.

Potential exclusions:

- Exclude the patients with a stated rationale for performing CPR, even if they have consented to the DNAR policy, from the denominator.
- Exclude such cases in which the status of consenting to DNAR policy has changed after discharge from the ICU from the denominator.

Data source and collection methods: Electronic medical record data. All records of the eligible patients during the ICU stay. (Physician records, nurse records, progress notes, etc.)

**Structure indicators**

1. **Use of standardized pain measurement scales**
2. **Use of standardized dyspnea measurement scales**
3. **Use of standardized thirst measurement scales**
4. **End-of-life specific symptom management care protocols or order sets**
5. **Availability of a palliative care team**
6. **Availability of a specialized psychiatric team**
7. **A flexible visitation policy**
8. **Rooms with privacy for discussions between healthcare providers and family members**
9. **System to provide mental health care to patients and their families ***
10. **Leaflet for family members, including information on orientation to the ICU environment and delirium care**
11. **A "Critical Care Mediator for Inpatients" is in place ✝️**
12. **Regular opportunities for ICU staff to reflect on their end-of-life care experiences to support their emotional well-being**
13. **Access to palliative care specialists and other professionals to discuss ethical issues related to treatment**

***** The system for providing mental health care to patients/families refers to whether there are resources and referral flows available within the hospital. The implementers include clinical psychologists, clinical religious workers, and psychiatric liaison teams.

**✝️** A "Critical Care Mediator for Inpatients" is a mediator who supports critically ill patients and their families from the time a patient is admitted. Mediators are personnel who coordinate between the medical staff and the family, and carefully connect the patient's family to the medical staff to resolve any concerns or questions they may have.

Data source and collection methods: Facility survey (interviews or questionnaires)

〈List of abbreviations〉

NRS; Numeric Rating Scale

CPOT; The Critical-Care Pain Observation Tool

BPS; Behavioral Pain Scale

GCS; Glasgow Coma Scale

DNAR; Do Not Attempt Resuscitation

CPR; Cardiopulmonary Resuscitation

ACP; Advance Care Planning
