# Supplementary material for: Development of quality indicators for palliative care in intensive care units and pilot testing them via electronic medical record review
Source: J Intensive Care. 2024 Jan 9;12:1. doi: 10.1186/s40560-023-00713-z (PMC10775577; doi:10.1186/s40560-023-00713-z)
Supplement: Supplementary file 3 — Additional file 3. Table. Inter-rater reliability verification. [file 40560_2023_713_MOESM3_ESM.docx]

Additional file 3. Table. Inter-rater reliability verification

|  | Indicators | Difference * | Cohen's kappa † | 95% confidence interval |
| --- | --- | --- | --- | --- |
| **Process** | |  |  |  |
| 1 | Regular pain assessment | 0.006 | . |  |
| 2 | Appropriate pain management | 0.136 | . |  |
| 3 | Reassessment of pain after treatment and/or management | 0.016 | . |  |
| 4 | Regular delirium assessment ‡ | 0.057 | . |  |
| 5 | Assessment of the patient's psychological distress | . | 0.79 | 0.51-1.00 |
| 6 | Assessment of social support needs | . | 1 | 1 |
| 7 | Assessment of the patient's spiritual and cultural practices | . | 1 | 1 |
| 8 | Identification of the patient's advance directive and ACP for treatment | . | 1 | 1 |
| 9 | Conduct of an interdisciplinary family conference on palliative care | . | 1 | 1 |
| 10 | Transmission of key information regarding palliative care following ICU transfer | . | 0.76 | 0.52-1.00 |
| 11 | Assessments of psychological distress of family members | . | 1 | 1 |
| 12 | Documentation of the medical process regarding end-of-life decisions | . | 0.87 | 0.61-1.00 |
| 13 | Modification of medical care for it to be in concordance with the goals of care for patients at the end of life | . | 1 | 1 |
| **Outcome** | |  |  |  |
| 14 | Patient pain-free in the last 24 h of life | . | 0.87 | 0.61-1.00 |
| 15 | Avoid performing CPR when the patient does not want | . | 0.79 | 0.51-1.00 |
| **Overall (QI 5〜15)** ‡ | | . | 0.92 | 0.87-0.97 |

ACP, Advance Care Planning; CPR, Cardiopulmonary Resuscitation

* In QI1-4, the mean of the difference in the percentage of implementation was calculated.

† In QI 5-15, Cohen's kappa coefficients and 95% confidence intervals were calculated for each item.

‡ Cohen's kappa coefficient and 95% confidence interval for QI 5-15 overall were calculated.
